# Correlation of blood bone turnover biomarkers and Wnt signaling antagonists with AS, DISH, OPLL, and OYL

**DOI:** 10.1186/s12891-017-1425-4

**Published:** 2017-02-02

**Authors:** Chi-Chien Niu, Song-Shu Lin, Li-Jen Yuan, Lih-Huei Chen, Chuen-Yung Yang, An-Ni Chung, Meng-Ling Lu, Tsung-Ting Tsai, Po-Liang Lai, Wen-Jer Chen

**Affiliations:** 1 Department of Orthopaedic, Chang Gung Memorial Hospital, Linkou, Taiwan; 2 College of Medicine, Chang Gung University, Taoyuan, Taiwan; 3 Bone and Joint Research Center, Chang Gung Memorial Hospital, Linkou, Taiwan; 4 Department of Orthopaedic, Chang Gung Memorial Hospital, Taoyuan, Taiwan; 5 Department of Orthopaedic Surgery, Chang Gung Memorial Hospital, No 5, Fu-Hsing Street 333, Taoyuan, Taiwan

**Keywords:** Wnt inhibitor, OPLL, OYL, AS, DISH

## Abstract

**Background:**

Wnt signaling plays an important role in development and maintenance of many organs and tissues. The most-studied secreted Wnt inhibitors are sclerostin (SOST), Dickkopf-related protein 1 (DKK-1), and secreted frizzled related protein 1 (SFRP-1) which play important roles in bone turnover. The present study investigated the relationship between serum Wnt inhibitors and diseases with excessive ossification structures, such as ossification of posterior longitudinal ligament (OPLL), ankylosing spondylitis (AS), diffuse idiopathic skeletal hyperostosis (DISH), and ossification of yellow ligament (OYL).

**Methods:**

Twenty-five patients with AS, DISH, OPLL, or OYL were recruited in this study. Fasting peripheral blood samples were collected from all patients and nine controls. Various biomarkers of bone turnover including osteocalcin (OSC), osteoprotegerin (OPG), SFRP-1, DKK-1, and SOST were investigated.

**Results:**

Our data showed that serum levels of OSC were higher, but Dkk-1 levels were lower in AS, DISH, OPLL, and OYL patients than those in the controls. Serum levels of SFRP-1 were significantly higher in DISH patients than those in the controls. Serum levels of SOST were significantly higher in DISH and OPLL patients than both levels in the controls. Serum levels of OPG were lower in AS patients than those in the controls. Serum levels of OSC were higher in the OPLL patients than those in the AS patients. Serum levels of DKK-1, SFRP-1, SOST, and OPG were not significantly different between the different disease groups.

**Conclusions:**

In this exploratory study, both OSC and DKK-1 levels are correlated with the clinical conditions associated with excessive ossification, indicating that blood OSC and DKK-1 levels may serve as diagnostic biomarkers for AS, DISH, OPLL, and OYL. These findings may also help discover potential drug therapies for management of these diseases in the future.

## Background

Spondyloarthropathies are inflammatory disorders involving peripheral joints, sacroiliac joints, diffuse spine involvement, and some extra-articular features [1–3]. Ankylosing spondylitis (AS) presents with common and severe spine involvement. Earlier reports suggested that AS patients have low trabecular bone mineral density (BMD) in the spine [4]. Patients with AS are at high risk of osteoporosis and vertebral fractures [5]. Diffuse idiopathic skeletal hyperostosis (DISH) is also an ossifying diathesis of unknown etiology, characterized by flowing calcification and ossification on the anterolateral aspect of contiguous vertebral bodies with no involvement of apophyseal joints and sacroiliac joints [6]. Ossifying posterior longitudinal ligament (OPLL) is a condition of abnormal calcification of the posterior longitudinal ligament. The etiology of OPLL has not been fully clarified [7]. OPLL seems to occur and develop as a result of systemic and local factors in combination with a genetic abnormality [8, 9]. Ossification of the yellow ligament (OYL) is characterized by progressive ectopic bone formation in the spinal ligaments. Even though the pathogenesis of OYL is unclear, mechanical stress on the yellow ligament has been identified as a main contributor [10]. The OPLL and OYL of spine have an unknown etiology and are troublesome diseases in surgical treatment. Combinations of varying degrees of spondylosis and/or OPLL, and OYL contribute to thoracic and lumbar neural compression in North Americans [11].

Excessive ossification of the tissue around the spine, albeit in different regions, is a common characteristic of these aforementioned spondyloarthropathies. The excessive ossification causes two serious pathologic problems: loss of motion occurs between spine segment(s), and the space-occupying-lesion compresses the neurological structure. These pathologies always have multiple foci that are distributed along the spine. OPLL has been reported to be associated with DISH [12, 13], AS [14], and other spondyloarthropathies [15]. Clinically, DISH and OPLL, DISH and OYL, OPLL and OYL, and AS and OYL have indeed been reported to coexist in the same patients. Because of this overlap, we sought to investigate whether the pathophysiology of these lesions are similar but show various degrees of activity, or have totally different mechanisms. It might be possible to devise methods for reversing the progression of these diseases and preventing the poor prognosis at the late stage once the mechanisms of the excessive ossification in these diseases are clarified.

Few reports describe the relationships between AS, DISH, OPPL, OYL, and the Wnt pathway. Wnt signaling plays an important role in development and maintenance of many organs and tissues [16]. Although Wnt signals through several pathways to regulate cell growth, differentiation, function, and death, the Wnt/β-catenin or canonical pathway appears to be particularly important for bone biology [17, 18]. The Wnt/β-catenin pathway is an osteogenic pathway. The most-studied secreted Wnt inhibitors are sclerostin (SOST), dickkopfs (DKKs), and secreted frizzled related proteins (SFRPs), which likely play important roles in bone turnover [19]. SOST, a secreted glycoprotein of osteocytes, is thought to directly bind to lipoprotein receptor-related proteins (LRPs) and prevent Wnt ligand binding [20]. SFRP-1 is thought to competitively inhibit binding of Wnts to the LRP/Frzled complex by acting as decoy receptors [21]. Similar to SOST, DKK-1 is a secreted antagonist of Wnt/β-catenin signaling which also functions by binding to the LRP5/6 co-receptor. These complexes are rapidly endocytosed, and can prevent the Wnt-LRP interaction [22, 23]. Osteoblasts produce osteoprotegerin (OPG), which is a soluble decoy receptor for receptor activator of nuclear factor κB ligand (RANKL) [24]. OPG inhibits osteoclastogenesis by blocking the RANKL–RANKL receptor interaction. The activation of the canonical Wnt pathway in osteoblasts suppresses bone resorption through upregulation of OPG expression and downregulation of RANKL expression [25].

Human global gene expression analyses have identified DKK-1 as a bone mineral density (BMD)-associated gene in postmenopausal women, and serum DKK-1 levels are inversely associated with BMD in osteoporosis [23, 26]. DKK-1 blockade reverses both the bone-destructive pattern of an inflammatory arthritis model and the bone-forming pattern of osteoarthritis [27], whereas DKK-1 blockade promotes ankylosis of sacroiliac joints in an experimental model [28]. Recent studies have shown the patients with AS [29] and DISH [30] have lower serum levels of DKK-1 than controls. This finding suggests that the excessive ossification has some correlation with lower serum levels of DKK-1, which, in turn, may contribute to the pathogenesis of these diseases.

In this study, patients with a diagnosis of DISH, AS, OPLL, or OYL, as assessed by radiographic and magnetic resonance image (MRI) studies were enrolled. Various biomarkers of bone turnover and Wnt/β-catenin signaling antagonists including OSC, OPG, SFRP-1, DKK-1, and SOST were quantified. We detected the biomarkers in serum and evaluated their association with the spinal involvement in DISH, AS, OPLL, and OYL.

## Methods

### Patient’s collection

Consecutive patients with features of DISH, AS, OPLL, or OYL at the Chang Gung Memorial Hospital were enrolled in this study and written informed consent was obtained from enrolled patients. Patients were diagnosed with one of the aforementioned diseases based on the presence of clinical features by radiographic and/or magnetic resonance imaging (MRI) scans. The clinical features of ankylosing spondylitis (AS) were: the deteriorated stiffness of whole spine and fixed in a kyphotic deformity in the end stage; and decreasing range of motion of the major joints, like hips and sacroiliac joints, by the progressive ossification of the ligament or tendinous insertion into bones. Diffuse idiopathic skeletal hyperostosis (DISH) was characterized by flawing calcification and ossification of the anterolateral aspect of contiguous vertebral bodies (3 and more than 3), with ossification of extra spinal sites (osteophyte like). OPLL was diagnosed based on ossification of the longitudinal ligament seen in the radiographic studies, including lateral spine radiograms and computed tomography (CT), extended from C1 to C7. The ossification of yellow ligament (OYL) was defined by ossification of the ligmentum flavum, which was located on the posterior and lateral sides of the dural sac. Patients without a diagnosis of OPLL, OYL, AS, and DISH served as controls.

### Serum biomarker measurement

Fasting peripheral blood samples (10 ml) were collected from all patients at the time of diagnosis and from controls. After coagulation, serum samples were centrifuged and stored at −80 °C until analysis. Enzyme-linked immunosorbent assays (ELISA) were used to measure the levels of serum OSC (Osteocalcin Instant ELISA, Affymetrix eBioscience, Austria), DKK-1 (Quantikine ELISA, R&D system, USA), sclerostin (Quantikine ELISA, R&D system, USA), osteoprotegerin (OPG ELISA, abcam, UK), and secreted frizzled-related protein 1 (SFRP-1 ELISA, USCN, USA) in patient and control samples and were performed according to the manufacturer’s protocols.

### Statistical analysis

In this exploratory study, data are represented using the mean ± SD. Differences in levels of biomarkers between patients and controls were analyzed using Mann-Whitney test. The differences between the disease groups with respect to these serum markers were also assessed via Mann-Whitney test. The level of significance was set at 0.05. The statistical analyses were performed using the Excel or SPSS software package (Version 12.0, Chicago, IL). Log_10_ (parameter concentration) model was used to put all parameters into one figure in this study.

## Results

### Characteristics of the patients and controls

Six patients were diagnosed with AS (Male: 5, Female: 1; age 54.0 ± 19.1 years), and 8 patients with DISH (Male: 7, Female: 1; age 60.4 ± 7.2 years). OPLL was found in 8 patients (Male: 5, Female: 3; age 58.6 ± 9.2 years), 3 patients were diagnosed with OYL (Male: 1, Female: 2; age 62.7 ± 19.1 years), and there were 9 controls (Male: 2, Female: 7; age 55.9 ± 15.9 years). There were no significant differences in terms of age between the patients and the controls.

### Bone turnover factors and Wnt signaling antagonists in patients with AS and controls

**Fig. 1:**
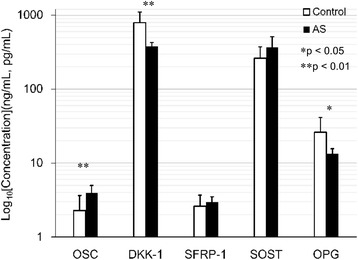
Bone turnover factors and Wnt signaling antagonists in patients with AS and controls. Serum levels of OSC (**p < 0.01) were higher but those of DKK-1 (**p < 0.01) and OPG (*p < 0.05) were lower in the patients with AS than in the controls. Serum levels of SFRP-1and SOST were not significantly different between the AS patients and controls. (OSC, SFRP-1, and OPG: ng/ml; Dkk-1, SOST: pg/mL)

Figure 1 and Table 1 summarize the circulating levels of OSC, Dkk-1, SFRP-1, SOST, and OPG in the patients with AS and the controls. Serum levels of OSC were higher in the patients with AS than in the controls (**p < 0.01). Serum levels of DKK-1 (**p < 0.01) and OPG (*p < 0.05) were lower in the patients with AS than in the controls. Serum levels of SFRP-1and SOST were not significantly different between the AS patients and controls.

**Table 1:** Levels of OSC, Dkk-1, SFRP-1, SOST, and OPG in the patients with AS and controls

	OSC, ng/ml	Dkk-1, pg/ml	SFRP-1, ng/ml	SOST, pg/ml	OPG, ng/ml
AS (*n* = 6)	3.96 ± 1.03	379.8 ± 48.1	2.99 ± 0.51	368.7 ± 143.9	13.4 ± 2.3
Control (*n* = 9)	2.28 ± 1.37	792.5 ± 308.6	2.61 ± 1.08	261.1 ± 111.4	26.1 ± 15.3

### Bone turnover factors and Wnt signaling antagonists in patients with DISH and controls

**Fig. 2:**
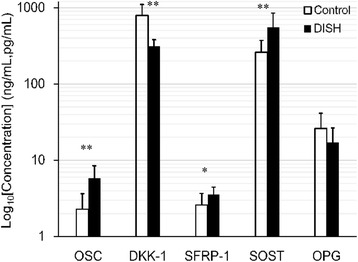
Bone turnover factors and Wnt signaling antagonists in patients with DISH and controls. Serum levels of OSC (**p < 0.01), SFRP-1 (*p < 0.05) and SOST (**p < 0.01) were higher in the patients with DISH than in the controls. Serum levels of DKK-1 (**p < 0.01) were lower in the patients with DISH than in the controls. There was no significant difference in OPG levels between the DISH patients and controls. (OSC, SFRP-1, OPG: ng/mL; DKK-1, SOST: pg/mL)

Figure 2 and Table 2 summarize the circulating levels of OSC, Dkk-1, SFRP-1, SOST, and OPG in patients with DISH and the controls. Serum levels of OSC were higher in the patients with DISH than in the controls (**p < 0.01). Serum levels of DKK-1 were lower in the patients with DISH than in the controls (**p < 0.01). Serum levels of SFRP-1 (*p < 0.05) and SOST (**p < 0.01) were higher in the patients with DISH than in the controls. There was no significant difference in OPG levels between the DISH patients and controls.

**Table 2:** Levels of OSC, Dkk-1, SFRP-1, SOST, and OPG in the patients with DISH and controls

	OSC, ng/ml	Dkk-1, pg/ml	SFRP-1, ng/ml	SOST, pg/ml	OPG, ng/ml
DISH (*n* = 8)	5.84 ± 2.66	311.9 ± 68.5	3.69 ± 0.86	550.6 ± 302.2	17.1 ± 9.5
Control (*n* = 9)	2.28 ± 1.37	792.5 ± 308.6	2.61 ± 1.08	261.1 ± 111.4	26.1 ± 15.3

### Bone turnover factors and Wnt signaling antagonists in patients with OPLL and controls

**Fig. 3:**
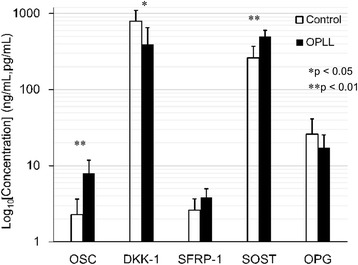
Bone turnover factors and Wnt signaling antagonists in patients with OPLL and controls. Serum levels of OSC (**p < 0.01) and SOST (**p < 0.01) were higher but those of DKK-1 (*p < 0.05) were lower in the patients with OPLL than in the controls. There was no significant difference in SFRP-1 and OPG levels between the OPLL patients and controls. (OSC, SFRP-1, OPG: ng/mL; Dkk-1, SOST: pg/mL)

Figure 3 and Table 3 summarize the circulating levels of OSC, Dkk-1, SFRP-1, SOST, and OPG in patients with OPLL and controls. Serum levels of OSC (**p < 0.01) and SOST (**p < 0.01) were higher in the patients with OPLL than in the controls. Serum levels of DKK-1 were lower in the patients with OPLL than in the controls (*p < 0.05). There was no significant difference in SFRP-1 and OPG levels between the OPLL patients and controls.

**Table 3:** Levels of OSC, Dkk-1, SFRP-1, SOST, and OPG in the patients with OPLL and controls

	OSC, ng/ml	Dkk-1, pg/ml	SFRP-1, ng/ml	SOST, pg/ml	OPG, ng/ml
OPLL (*n* = 8)	7.95 ± 3.91	395.8 ± 260.1	3.82 ± 1.17	499.4 ± 104.1	17.2 ± 8.2
Control (*n* = 9)	2.28 ± 1.37	792.5 ± 308.6	2.61 ± 1.08	261.1 ± 111.4	26.1 ± 15.3

### Bone turnover factors and Wnt signaling antagonists in patients with OYL and controls

**Fig. 4:**
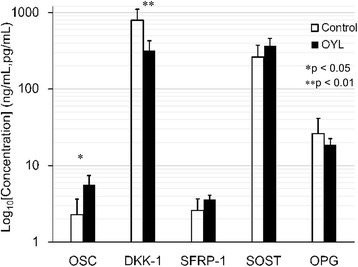
Bone turnover factors and Wnt signaling antagonists in patients with OYL and controls. Serum levels of OSC (*p < 0.05) were higher but those of DKK-1 (**p < 0.01) were lower in the patients with OYL than in the controls. No significant differences in SFRP-1, SOST and OPG levels in the OYL patients and controls were detected. (OSC, SFRP-1, OPG: ng/mL; Dkk-1, SOST: pg/mL)

Figure 4 and Table 4 summarize the circulating levels of OSC, DKK-1, SFRP-1, SOST, and OPG in patients with OYL and controls. Serum levels of OSC were higher in the patients with OYL than in the controls (*p < 0.05). Serum levels of DKK-1 were lower in the patients with OYL than in the controls (**p < 0.01). No significant differences in SFRP-1, SOST and OPG levels in the OYL patients and controls were detected.

**Table 4:** Levels of OSC, Dkk-1, SFRP-1, SOST, and OPG in the patients with OYL and controls

	OSC, ng/ml	Dkk-1, pg/ml	SFRP-1, ng/ml	SOST, pg/ml	OPG, ng/ml
OYL (*n* = 3)	5.62 ± 1.78	316.1 ± 112.1	3.61 ± 0.49	368.9 ± 91.4	18.7 ± 3.79
Control (*n* = 9)	2.28 ± 1.37	792.5 ± 308.6	2.61 ± 1.08	261.1 ± 111.4	26.1 ± 15.3

### Bone turnover factors and Wnt signaling antagonists in patients with different diseases

Table 5 summarizes the data for bone turnover factors and Wnt signaling antagonists in patients with different diseases. Serum levels of OSC were higher in the OPLL patients than those in AS patients (**p < 0.01). Serum levels of DKK-1, SFRP-1, SOST, and OPG were not significantly different between the disease groups (*p* > 0.05).

**Table 5:** Levels of OSC, Dkk-1, SFRP-1, SOST, and OPG in the patients with different diseases

	OSC, ng/ml	Dkk-1, pg/ml	SFRP-1, ng/ml	SOST, pg/ml	OPG, ng/ml
AS	3.96 ± 1.03	379.8 ± 48.1	2.99 ± 0.51	368.7 ± 143.9	13.4 ± 2.3
DISH	5.84 ± 2.66	311.9 ± 68.5	3.69 ± 0.86	550.6 ± 302.2	17.1 ± 9.5
OPLL	7.95 ± 3.91	395.8 ± 260.1	3.82 ± 1.17	499.4 ± 104.1	17.2 ± 8.2
OYL	5.62 ± 1.78	316.1 ± 112.1	3.72 ± 0.46	368.9 ± 91.4	18.7 ± 3.79

## Discussion

Ankylosing spondylitis (AS) is a chronic inflammatory disease characterized by inflammation of the spine and can lead to bone erosion, new bone formation, and ankylosis of the spine. In patients with AS, after the initial destructive changes driven by inflammation of the joint are observed, blockade of DKK-1 relieves Wnt signaling from DKK-1-mediated suppression and induces bone formation, as mirrored by the growth of osteophytes [27]. However, whether the serum levels of DKK-1 are significantly altered in AS patients remains an unresolved issue. Previous studies suggested that the DKK-1 levels were lower [29], higher [31], or similar [32] in the AS patients compared to the levels in healthy controls. Kwon et al. [29] showed that serum DKK-1 levels were significantly lower, but that OSC levels were significantly higher in AS patients than those in the controls. Daoussis et al. [31] suggested that the inhibitory effect of DKK-1 in sera from AS patients on Wnt pathway activation was negligible, in contrast to the clear inhibitory effect of DKK-1 in sera from healthy donors. In the present study, we measured DKK-1 levels in patients with AS and found that DKK-1 levels are significantly lower in AS patients than those in controls. In addition, the level of OSC was significantly higher in AS patients than in controls (Fig. 1). Consistent with the model of Diarra et al. [27] and Kwon et al. [29], we would expect the serum DKK-1 levels in patients with AS to be suppressed but the levels of OSC to increase during osteophyte formation. Low serum levels of DKK-1 in AS patients suggest that Wnt signaling is activated. Wnt activation can induce new bone formation in arthritic joints and also allow the growth of osteophytes.

Canonical Wnt proteins induce OPG expression, which blocks the receptor activator of nuclear factor kappa b ligand (RANKL)-mediated bone resorption [33]. Franck et al. [34] and Taylan et al. [32] showed that OPG levels were significantly lower in AS patients compared to healthy subjects. However, some studies revealed elevated OPG levels in AS patients compared to controls [29, 35]. Our data showed that the OPG level was significantly lower in AS patients than in controls (Fig. 1). We surmise that the formation of osteophytes is directly controlled by Wnt signaling. The proliferation and differentiation of mesenchymal tissue that results in the formation of osteophytes can occur independently of the RANKL–OPG system, because inhibition of OPG does not impair osteophyte formation [27].

DISH is characterized by new bone formation, calcification, and ossification of the anterior longitudinal ligament of the spine and various extra spinal ligaments [6]. Two recent studies investigated the relationship between serum DKK-1 and DISH [30, 36]. Senolt et al. [30] showed that the levels of total serum DKK-1 were significantly lower in patients with DISH than in healthy controls. Importantly, low serum levels of DKK-1 were associated with more severe spinal involvement in DISH [30]. However, Aeberli et al. [36] suggested that DKK-1 levels of DISH patients were not different from healthy controls and hence development of DISH is unlikely to be dependent on the Wnt signaling inhibitor DKK-1 [36]. Consistent with the model of Senolt et al. [30], we suggest that the lower circulating DKK-1 level and higher OSC level in patients with DISH reflect the bone anabolic phenotype of the disease (Fig. 2).

SOST plays a critical role in bone and mineral metabolism. Kashii et al. [37] showed that systemic secretion of SOST increases with higher bone mass in men with OPLL and that serum SOST levels negatively correlate with DKK1 levels in men with OPLL. Higher serum SOST levels are counterbalanced by underproduction of DKK1 [37]. In the present study, we found that the DKK-1 levels were significantly lower in patients with OPLL than those in controls. In addition, the levels of SOST were significantly higher in patients than those in controls (Fig. 3). Consistent with the model of Kashii et al. [37], we suggest that the higher serum SOST levels are counterbalanced by underproduction of DKK1. Fifty percent of the patients with DISH also have OPLL, and OPLL is thought to be a subtype of DISH [38]. Our study suggests that changes in serum DKK-1 levels observed in patients with DISH may also represent an adaptive response to changes in serum SOST levels (Fig. 2).

SFRP-1 inhibits Wnt signaling either by binding to Wnts [21] or the LRP5/6 complex [39] to prevent the Wnt - LRP5/6 interaction. Consistent with the model of Taylan et al. [40], our data showed that the level of SFRP-1 was similar between the AS patients and controls (Fig. 1). Because SOST and SFRP-1 both act as extracellular antagonists of the Wnt/β-catenin signaling pathway, we suggest that SOST and SFRP-1 play similar roles in patients with DISH. Our data showed the higher levels of SFRP-1 and SOST and lower levels of DKK-1 in patients with DISH (Fig. 2) than those in controls. Similar to SOST, elevated serum SFRP-1 levels may be counterbalanced by underproduction of DKK-1.

Sugimori et al. [40] reported that the concentrations of OSC were significantly higher in patients with OPLL than those in controls. Thus, the serum concentration of OSC may reflect the activity of general ectopic bone formation in patients with OPLL [40]. However, Ishihara et al. [41] and Matsui et al. [42] reported that serum OSC levels were similar in persons with OPLL and in control groups. In the present study, we found that the OSC levels were significantly higher in patients than those in controls (Fig. 3). Consistent with the model of Sugimori et al. [40], we found that the serum DKK-1 levels in patients with OPLL are suppressed, whereas the levels of OSC are increased, during osteophyte formation.

OYL is characterized by progressive ectopic bone formation in the spinal ligaments. Decreased DNA methylation in the promoter region of the Wnt5a and glial cell line-derived neurotrophic factor (GDNF) genes may promote the osteogenic ability of mesenchymal stem cells (MSCs) from patients with OYL [43]. However, the levels of bone turnover biomarkers, including OSC, OPG, SFRP-1, DKK-1, and SOST in serum have never been reported. Our data show that the serum DKK-1 levels in patients with OYL are suppressed but the levels of OSC are increased during osteophyte formation (Fig. 4).

Many previous studies showed that persons with OPLL, who often also have DISH, have a significantly higher BMD than control-groups [44, 45]. In this study, we mainly focused on the patients who were diagnosed with one type of the diseases associated with aberrant bone turnover. Although the serum levels of OSC, DKK-1, SFRP-1, SOST, and OPG were not significantly different between patients with one of these diseases (Table 5), we found that OSC levels were higher in OPLL (7.95 ± 3.91 ng/mL) than in AS (3.96 ± 1.03 ng/mL) patients (**p < 0.01). OPLL was found to be associated with more severe axial disease [15]. We hypothesize that the higher levels of OSC may be associated with the pathogenesis of OPLL progression.

We note that our study has several limitations. Our experiments were limited to a small number of samples in each group, it could be argued that the differences we observed were even more significant given the small size of the experimental groups. Because of the small number of patients, we could not find the correlations between serum parameters and clinical parameters of disease severity and progression in this study. We intend to address the relationship between disease severity and progression and serum biomarkers in a study with a larger set of patients in the future. In addition, OPLL has been associated with several diseases, including DISH ad AS. The serum markers in patients with more one of these bone turnover-related disease warrants further investigation. Recently, studies have demonstrated that dysfunctional MSCs contributes to many diseases. Considering that MSCs are a major source of osteoblasts, pathologic osteogenesis of MSCs in patients with AS, DISH, OPLL, and OYL are still controversial subjects.

## Conclusions

In this exploratory study, serum OSC levels were higher but DKK-1 levels were lower in AS, DISH, OPLL, and OYL patients than those in controls. Our results suggests that blood OSC and DKK-1 levels may serve as biomarkers for AS, DISH, OPLL, and OYL diagnosis. In addition, DISH and OPLL, DISH and OYL, OPLL and OYL, and AS and OYL have been noted to coexist in the same patients. Because of the disease overlapping, the present observations provide insights on the OSC and DKK-1 related mechanisms underlying the development of AS, DISH, OPLL, and OYL; and they ultimately may lead to potential drug therapies for management of these diseases. Future studies with larger subject cohorts are warranted to confirm the findings of this exploratory study.

## Abbreviations

AS: Ankylosing spondylitis
BMD: Bone mineral density
DISH: Diffuse idiopathic skeletal hyperostosis
DKK-1: Dickkopf-related protein 1
NF-*κ*B: Nuclear factor kappa b
OPG: Osteoprotegerin
OPLL: Ossification of posterior longitudinal ligament
OSC: Osteocalcin
OYL: Ossification of yellow ligament
RANKL: Receptor activator of nuclear factor κB ligand
SFRP-1: Secreted frizzled related protein 1
SOST: Sclerostin
